# Identification of Mutator-Derived Alternative Splicing Signatures of Genomic Instability for Improving the Clinical Outcome of Cholangiocarcinoma

**DOI:** 10.3389/fonc.2021.666847

**Published:** 2021-05-14

**Authors:** Zijing Lin, Jianping Gong, Guochao Zhong, Jiejun Hu, Dong Cai, Lei Zhao, Zhibo Zhao

**Affiliations:** ^1^ Department of Breast and Thyroid Surgery, the Second Affiliated Hospital of Chongqing Medical University, Chongqing, China; ^2^ Department of Hepatobiliary Surgery, The Second Affiliated Hospital, Chongqing Medical University, Chongqing, China; ^3^ Department of Hepatobiliary Surgery, The Second Affiliated Hospital & Centre for Lipid Research & Key Laboratory of Molecular Biology for Infectious Diseases (Ministry of Education), Chongqing Medical University, Chongqing, China

**Keywords:** cholangiocarcinoma, genomic instability, alternative splicing, immunotherapy, overall survival

## Abstract

**Background:**

Cholangiocarcinoma is an aggressive carcinoma with increasing incidence and poor outcomes worldwide. Genomic instability and alternative splicing (AS) events are hallmarks of carcinoma development and progression. The relationship between genomic instability, AS events, and tumor immune microenvironment remain unclear.

**Methods:**

The splicing profiles of patients with cholangiocarcinoma were obtained from The Cancer Genome Atlas (TCGA) spliceSeq database. The transcriptomics, simple nucleotide variation (SNP) and clinical data of patients with cholangiocarcinoma were obtained from TCGA database. Patients were divided into genomic unstable (GU-like) and genomic stable (GS-like) groups according to their somatic mutations. Survival-related differential AS events were identified through integrated analysis of splicing profiling and clinical data. Kyoto Encyclopedia of Genes and Genomes enrichment analysis was used to identify AS events occurring in genes enriched in cancer pathways. Pearson correlation was applied to analyze the splicing factors regulating AS events. CIBERSORT was used identify differentially infiltrating immune cells.

**Results:**

A prognostic signature was constructed with six AS events. Using this signature, the hazard ratio of risk score for overall survival is 2.362. For TCGA patients with cholangiocarcinoma, the area under the receiver operating characteristic curve is 0.981. CDK11A is a negative regulator of survival associated AS events. Additionally, the CD8+ T cell proportion and PD-L1 expression are upregulated in patients with cholangiocarcinoma and high splicing signatures.

**Conclusion:**

We provide a prognostic signature for cholangiocarcinoma overall survival. The CDK11A splicing factor and SLC46A1-39899-ES and IARS-86836-ES AS events may be potential targets for cholangiocarcinoma therapy. Patients with high AS risk score may be more sensitive to anti-PD-L1/PD1 immunotherapy.

## Introduction

Cholangiocarcinoma describes a group of carcinomas that occur in the biliary tree. Cholangiocarcinoma accounts for approximately 15% of all primary liver tumors and 3% of gastrointestinal cancers and the incidence of cholangiocarcinoma is increasing globally (1). In early stages cholangiocarcinoma is asymptomatic, leading to diagnosis in advanced stages and poor patient prognosis (2). The 5-year survival rate for patients with cholangiocarcinoma is 7–20% and tumor recurrence rates after resection remain disappointing (3). Therefore, there is an urgent need to find new biomarkers for cholangiocarcinoma diagnosis and prognosis.

Genomic instability is a driving factor of caner (4), and is associated with poor outcome in patients with cholangiocarcinoma (5, 6). To date, the molecular mechanisms of genomic instability in cholangiocarcinoma remain unclear. Recently, some microRNA (miRNA) 48 and long non-coding RNA (lncRNA) signatures associated with genomic instability have been identified. These signatures have efficiently predicted the outcome of ovarian cancer and breast carcinoma (7). However, whether alternative splicing (AS) events are associated with genomic instability remains unclear. However, whether genomic instability-related alternative splicing events predicted the outcome of cholangiocarcinoma remains unclear.

AS is a process through which exons within the same gene are expressed in different combinations, allowing a single gene to produce different proteins at different times and in different environments (8). The unbalanced expression of different isoforms of a single gene is recognized as contributing to the tumorigenesis and progression of numerous carcinomas (9). CD44v8-10 isoforms are upregulated in cholangiocarcinoma, and promote the proliferation of cholangiocarcinoma cells (10). Similarly, AS alternative events in WISP1v, Nek2B, ΔEX2TFF2, Foxp3Δ3, Δ133p53, PKM2, EP3−4, and AGR2vH are associated with the proliferation, migration, and invasion of cholangiocarcinoma cells (11).

In addition to affecting tumor cells, AS affects immune cells in the tumor microenvironment. Unbalanced ESRI1 isoforms are linked with infiltrating lymphocyte activity and patient survival (12). Similarly, AS events have been evaluated as predictive biomarkers for tumor immunotherapy in gastric cancer and squamous cell carcinoma (13, 14). Therefore, dysregulated AS events may serve as prognosis indicators and as potential therapy targets.

In this study, we describe a new prognostic signature model based on genomic instability derived AS events. Additionally, we explore the splicing factors that regulate the alternative splicing events recruited in our model. Furthermore, we analyzed the infiltrating immune cells correlated with this prognostic signature.

## Methods

### Data Collection

Transcriptomics, simple nucleotide variation, and clinical phenotype data of patients with cholangiocarcinoma (n = 36) were downloaded from The Cancer Genome Atlas (TCGA) database (https://tcga-data.nci.nih.gov/). AS data of patients with cholangiocarcinoma (n = 36) were downloaded from the TCGAspliceSeq database (http://bioinformatics.mdanderson.org). Percent splicing index values for AS events were applied to reflect the likelihood of each AS event.

### Identification of Genomic Instability Associated AS Events

**Figure 1:**
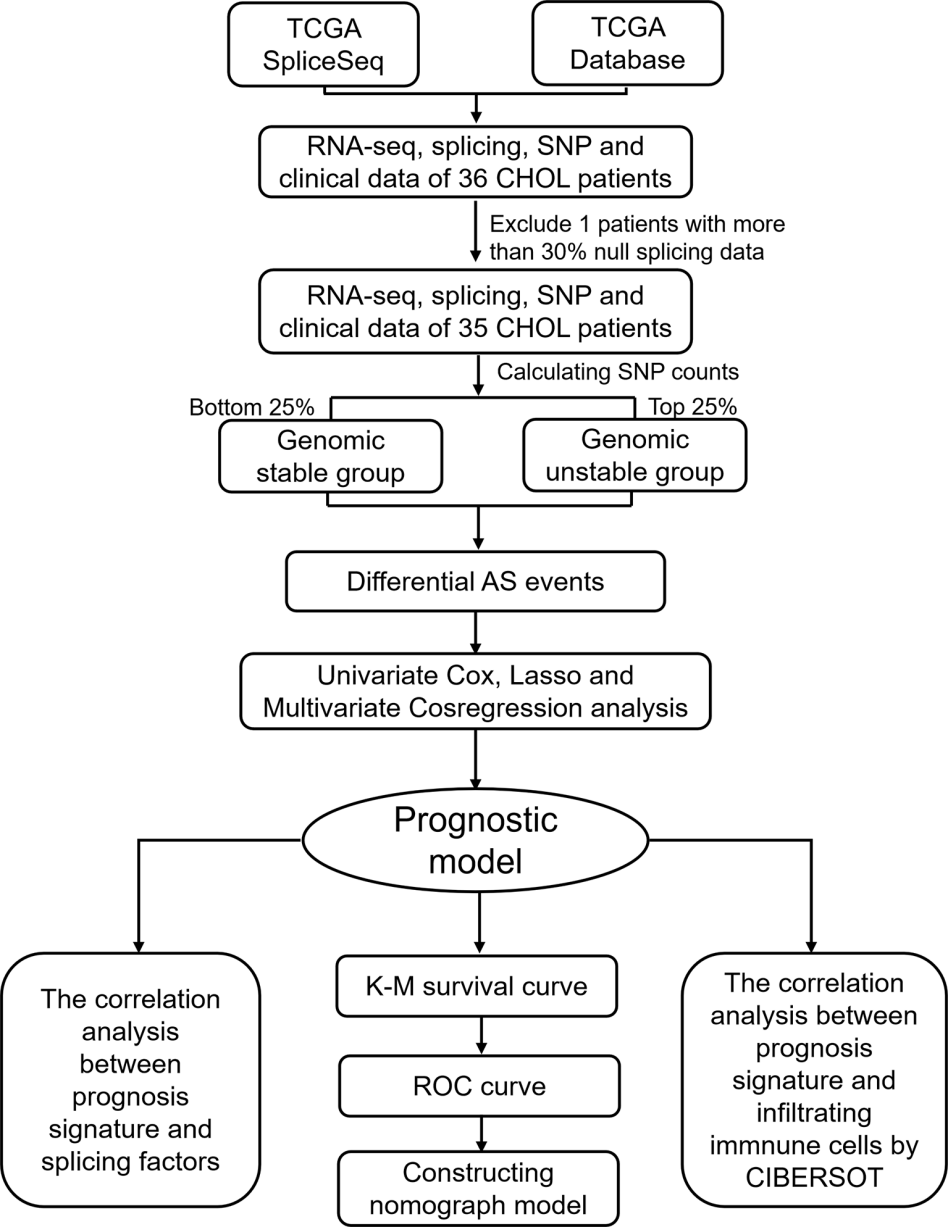
Flow diagram of the approach used in this study.

To identify genomic instability associated AS events, a mutator hypothesis-derived tumor genome computational framework combining Percent Spliced In (PSI) values of AS events and somatic mutation profiles was developed (**Figure 1**). This framework involved calculating the cumulative quantity of somatic mutations for each patient, and ranking patients in descending order of somatic mutation quantity. Then, the top 25% (n = 9) and the last 25% (n = 9) of patients were defined as genomic unstable (GU-like) and genomic stable (GS-like) group respectively. PSI values of AS events were compared between GU-like and GS-like groups with significance analysis of microarrays method. Differential AS events were defined as p < 0.05.

### Identification of Survival Associated AS Events and Construction AS Related Prognostic Signature

The AS events were visualized by Upset plot using UpSetR package (R version 4.0.3). Survival associated AS events were identified by univariate Cox regression using R software. AS events with P < 0.05 were used in further research. Lasso regression was performed to remove AS events having high correlation with each other. Multivariate Cox regression was performed to determine the prognostic value of each AS event. Finally, the prognostic signature model was constructed: $Riskscore=\Sigma_{i}^{n}PSIi*\beta i$ (β represents the regression coefficient of each event).

### Prognostic Signature Validation

Based on risk score, patients with cholangiocarcinoma were divided into two groups (high/low risk). K-M survival curve and Log-Rank tests were applied to compare overall survival (OS) between high and low risk groups. The ROC curve was applied to validate the predictive effect of the prognostic signature by calculating 5-year survival in R 4.0.3. Univariate Cox regression and Multivariate Cox regression were applied to calculate the hazard ratio (HR) of the high-risk score in OS.

### Correlation Between Splicing Factors and Survival Associated AS Events

Information about 404 splicing factors was obtained from a previous study (15). The expression of splicing factors was obtained from TCGA database. Pearson correlation analysis was performed to assess the relationship between splicing factor expression and the PSI value of AS events. Splicing factors and AS events with P < 0.05 and correlation coefficient > 0.7 were selected for building correlation plots with Cytoscape 3.7.2.

### Immune Cell Infiltration Analysis

CIBERSORT algorithm (http://cibersort.stanford.edu/), a computational framework providing immune cell type information from RNA profiles (16), was used to analyze the infiltering immune cells in cholangiocarcinoma tissue. Twelve cases with CIBERSORT P values were selected for the further analysis. These cases were divided into high-risk (n = 4) and low-risk (n = 8) groups based on their risk scores. The differential immune cell types between high- and low-risk groups were identified using the vioplot package of R 4.0.3.

## Results

### Clinical Characteristics and Integrated AS Events in Patients With Cholangiocarcinoma

**Figure 2:**
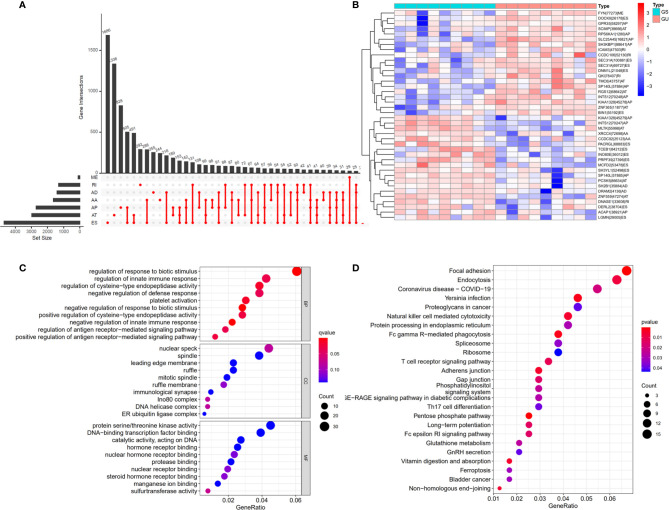
Differential alternative splicing (AS) events between genomic stable (GS-like) and unstable (GU-like) patients. **(A)** AS events and related genes in cholangiocarcinoma patients. **(B)** Differential AS events between GU-like and GS-like groups. **(C)** Bubble graph showing GO analysis of differential AS events. **(D)** Bubble graph showing KEGG analysis of AS events.

The workflow of this study is shown in **Figure 1**. In total, 36 patients with cholangiocarcinoma were enrolled in this study from TCGA. The baseline characteristics of enrolled patients are listed in **Table 1**. We identified 2146 alternate acceptor (AA) events in 1639 genes, 1846 alternate donor (AD) events in 1406 genes, 4877 alternate promoter (AP) events in 2700 genes, 5204 alternate termination (AT) events in 2965 genes, 9480 exon skipping (ES) events in 4768 genes, 105 mutually exclusive exon (ME) events in 103 genes, and 1856 retained intron (RI) in 1303 genes (**Figure 2A**).

**Table 1:** Characteristics of patients with cholangiocarcinoma from TCGA database.

Characteristics	No. of patients	%
Age		100.00
≥70	15	41.67
<70	21	58.33
Sex		100.00
Female	20	57.14
Male	26	74.29
Stage		100.00
I	19	54.29
II	9	25.71
III	1	2.86
IV	7	20.00
T category		100.00
T1	19	54.29
T2	12	34.29
T3	5	14.29
N category		100.00
N0	26	74.29
N1	5	14.29
unknown	5	14.29
M category		100.00
M0	28	80.00
M1	5	14.29
unknown	3	8.57

### Identification of Genomic Instability Related AS Events in Patients With Cholangiocarcinoma

To identify genomic instability related AS events, the cumulative quantities of somatic mutations in each patient were calculated and sorted in descending order. The top 25% (n = 9) and bottom 25% (n = 9) of patients were assigned to GU-like and GS-like groups, respectively. Then the AS events in patients in GU-like and GS-like groups were compared to identify differential AS events. In total, 644 differential AS events, with P values < 0.05, were identified. A heat map of the top 40 differential AS events was constructed (**Figure 2B**). Genes involved in the differential AS events were enriched 10 Gene Ontology (GO) and 25 KEGG pathways (**Figures 2C, D**).

### Construction of Survival-Associated AS Prognostic Model

**Figure 3:**
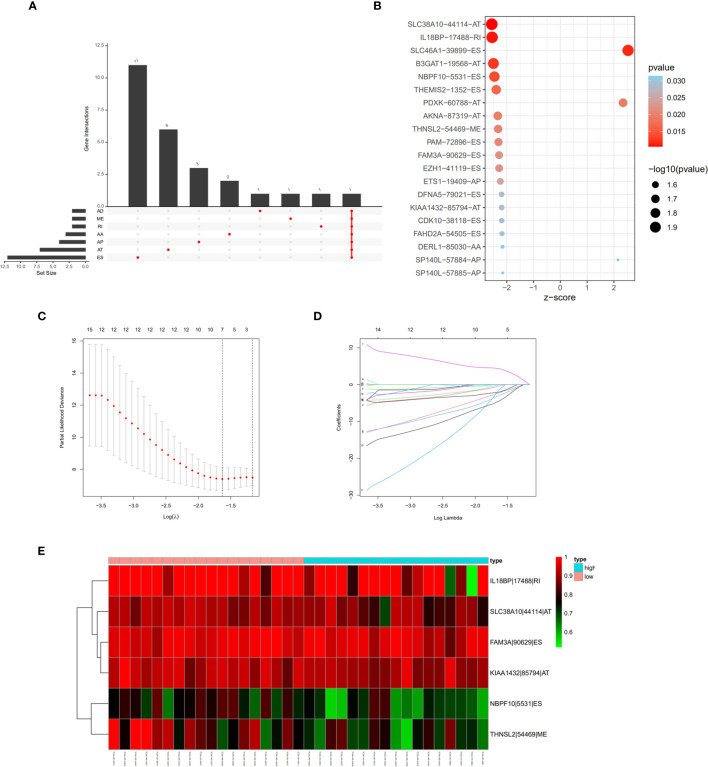
Prognostic signature construction based on differential alternative splicing (AS) events between genomic stable (GS-like) and unstable (GU-like) groups. **(A)** Overall survival related AS events in patients with cholangiocarcinoma distinguished by alternate acceptor (AA), alternate donor (AD), alternate promoter (AP), alternate termination (AT), exon skipping (ES), mutually exclusive exon (ME), and retained intron (RI) splicing mode. **(B)** Bubble graph of overall survival related AS events in cholangiocarcinoma with P values and z-scores. **(C, D)** cvFit and lambda graph of the lasso regression model. **(E)** Heat map showed the expression of AS events enrolled in our prognosis model.

Univariate cox regression analysis with P < 0.05 identified 26 AS events associated with cholangiocarcinoma progression (**Figures 3A, B**). Lasso regression analysis was performed on the 26 OS-related AS events to identify the events highly associated with cholangiocarcinoma (**Figures 3C, D**). Multivariate cox regression was applied to identify independent prognostic AS events. Finally, six AS events, SLC38A10-44114-AT, IL18BP-17488-RI, NBPF10-5531-ES, THNSL2-54469-ME, FAM3A-90629-ES, and KIAA1432-85794-AT, were identified as independent risk factors for OS in cholangiocarcinoma (**Figure 3E**). The risk score of each AS event was calculated (**Table 2**).

**Table 2:** Multivariate cox model.

id	coef	HR	HR.95L	HR.95H	pvalue
SLC38A10-44114-AT	-15.32	2.23E-07	1.61E-12	0.031	0.011
IL18BP-17488-RI	-6.09	0.002	1.23E-05	0.422	0.022
NBPF10-5531-ES	-17.24	3.24E-08	2.23E-12	0	<0.001
THNSL2-54469-ME	-15.04	2.95E-07	3.19E-11	0.003	0.001
FAM3A-90629-ES	-33.3	3.47E-15	3.14E-24	0	0.002
KIAA1432-85794-AT	-29.78	1.16E-13	2.78E-23	0	0.008

### Validation of the Prognostic Signature in Patients With Cholangiocarcinoma

**Figure 4:**
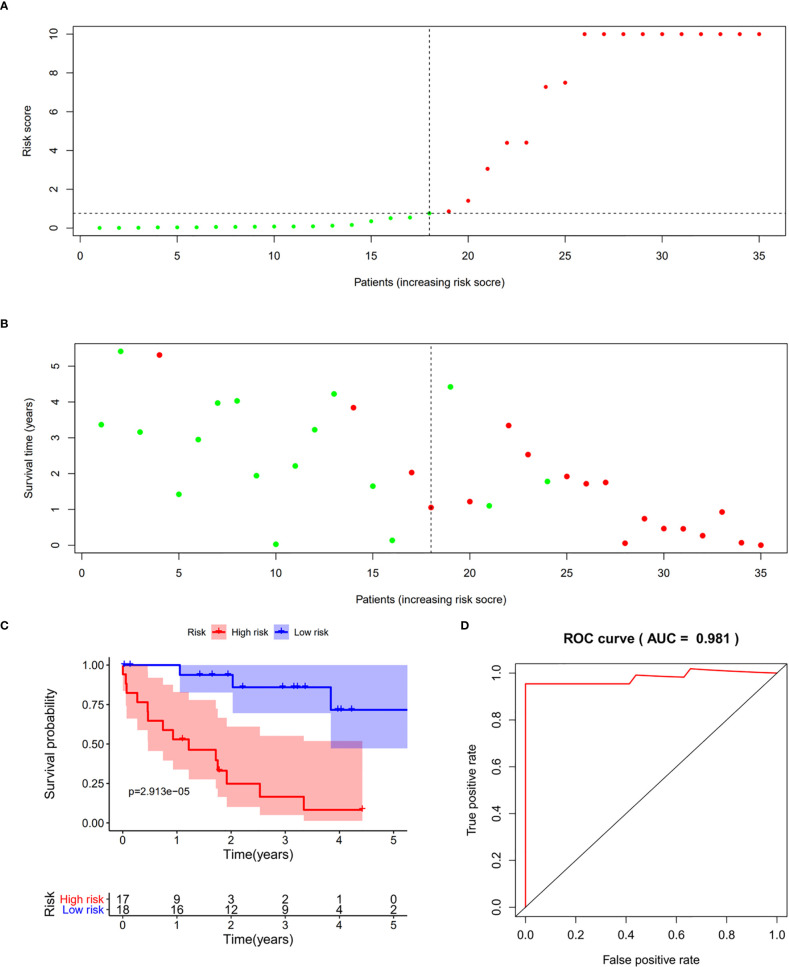
Prognostic model validation. **(A, B)** Survival time and survival status of patients with different risk scores. **(C)** Survival curve of patients with high and low risk scores. **(D)** Receiver operating characteristic curve of the prognostic model.

**Figure 5:**
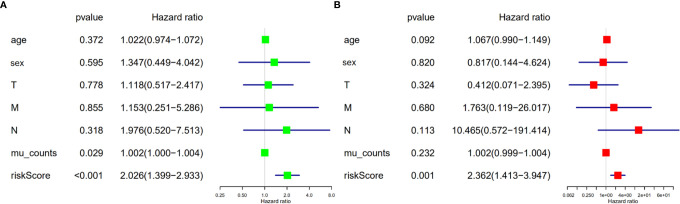
Risk score is an independent risk factor for overall survival in patients with cholangiocarcinoma. **(A)** Forest plot of univariate cox regression. **(B)** Forest plot of multivariate cox regression.

**Figure 6:**
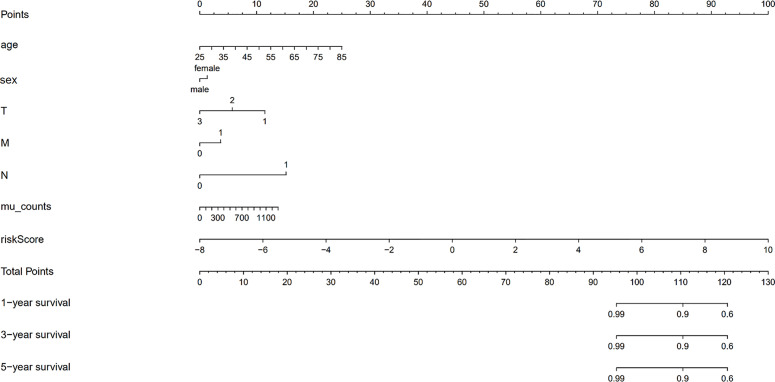
The nomograph model predicting 1-, 3-, and 5-year survival in patients with cholangiocarcinoma based on age, sex, TMN stage, mutation counts, and risk score.

We validated the predictive capability and efficiency of the prognostic signature. The risk score distribution curve showed that patients with cholangiocarcinoma and higher risk score have shorter survival time (**Figures 4A, B**). K-M survival curve analysis verified that patients with higher risk scores had poorer OS, P < 0.05 (**Figure 4C**). ROC curve (AUC = 0.981) analysis was performed to validate the efficiency of the risk score in OS prediction (**Figure 4D**) and the univariate and multivariate Cox regression HR values for OS were 2.026 and 2.362, respectively (**Figures 5A, B**). Collectively, these data demonstrate that the risk score of cancer related AS can be used to predict OS in patients with cholangiocarcinoma. In addition, we constructed a nomograph model predicting 1-, 3-, and 5-year survival of patients with cholangiocarcinoma (**Figure 6**).

### The Splicing Factors Regulating the Prognostic AS Events

**Figure 7:**
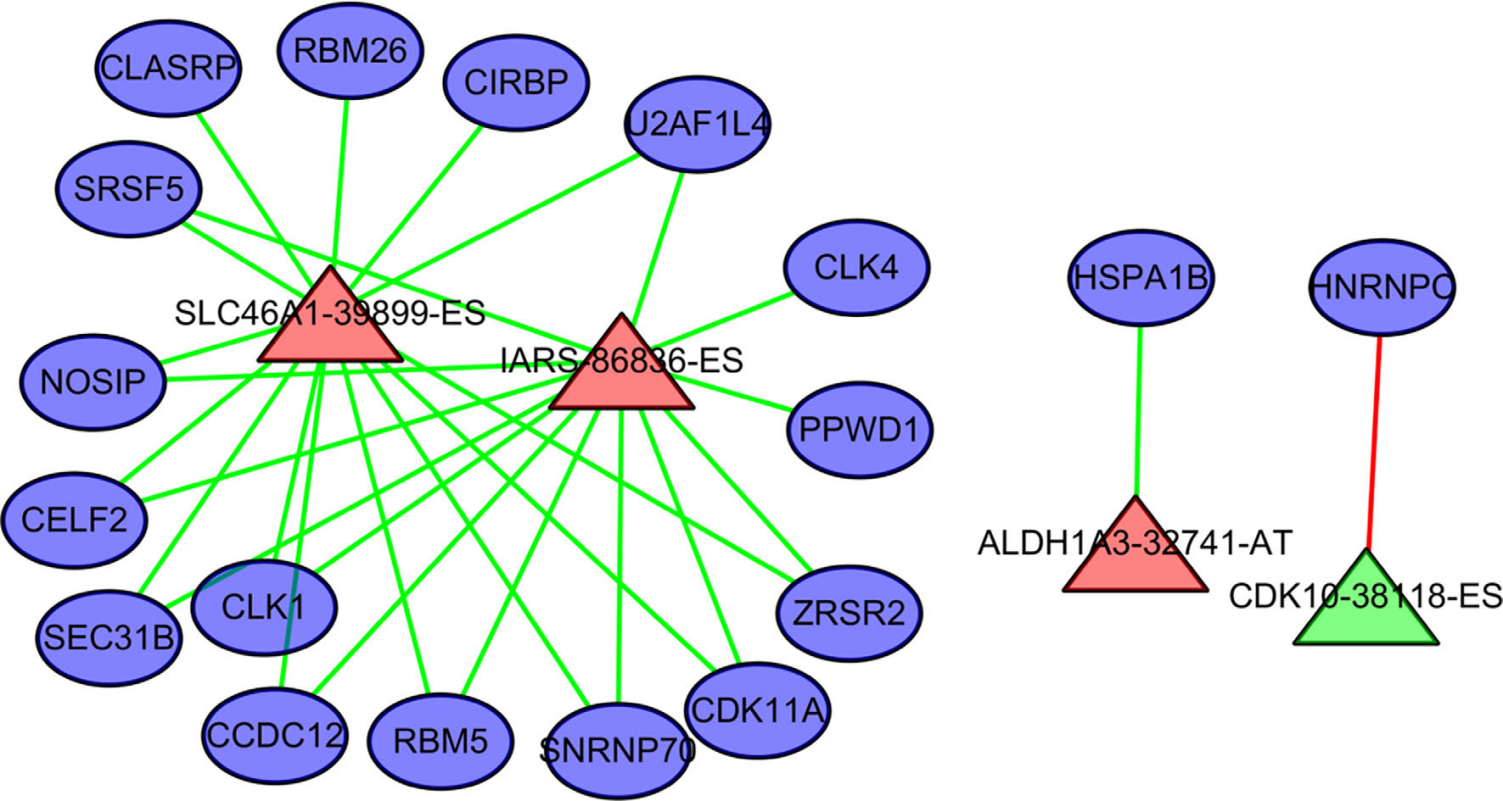
The regulation relationship between alternative splicing (AS) events and splicing factors. The ovals represent splicing factors. The red triangles represent AS events associated with poor outcome in cholangiocarcinoma. Green triangles represent AS events negatively associated with poor outcome in cholangiocarcinoma. The lines between AS events and splicing factors represent the relationship between them. Red lines represent upregulation. Green lines represent downregulation.

Four AS events and 18 splicing factors were identified using a Pearson’s correlation R value of > 0.7 and univariate cox regression P value of < 0.05 (**Figure 7**). Among these, SLC46A1-39899-ES, IARS-86836-ES, and ALDH1A3-32741-AT are upregulated AS events. The remained CDK10-38118-ES is a downregulated event.

**Figure 8:**
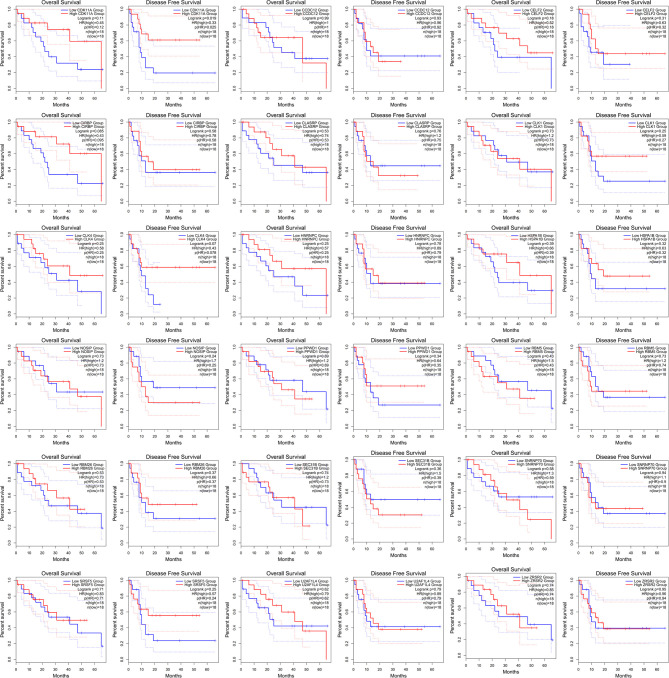
The receiver operating characteristic curve of alternative splicing (AS) signature related splicing factors in overall survival and disease-free survival in patients with cholangiocarcinoma.

HNRNPC is a core splicing factor that is positively correlated with down-regulated AS events. CCDC12, CLASRP, CLK4, RBM5, SEC31B, SRSF5, CIRBP, SNRNP70, ZRSR2, PPWD1, CLK1, CDK11A, NOSIP, U2AF1L4, RBM26, HNRNPC, HSPA1B, and CELF2 are core splicing factors that are negatively correlated with up regulated AS. We examined the relationship between these splicing factors and prognosis in patients with cholangiocarcinoma. Patients with higher CDK11A expression levels had higher disease-free survival rates (P = 0.023) than did patients with lower CDK11A expression levels. Patients with higher CIRBP expression levels had higher OS, but the associated P value is approaching insignificance (P=0.095, **Figure 8**).

### Revealing the Relationship Between Prognostic Signature and Tumor-Infiltrating Immune Cells in Tumor Microenvironment

**Figure 9:**
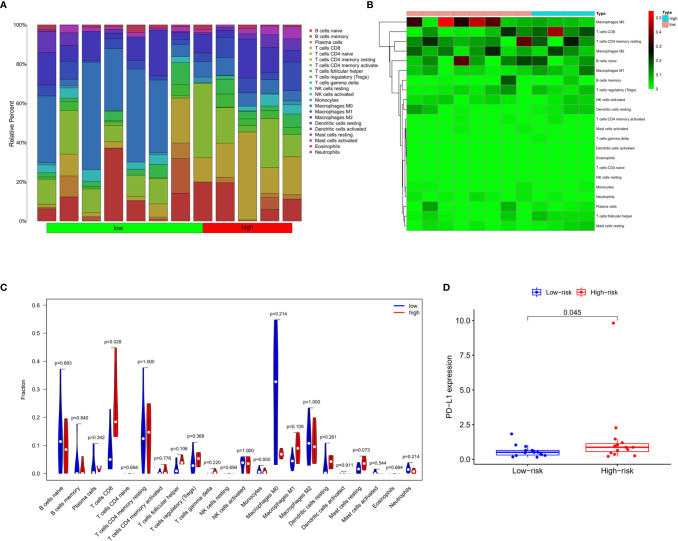
Infiltrating immune cells in cholangiocarcinoma. **(A)** Bar plot showing the proportion of 22 kinds of immune cells in patients with CIBERSORT value < 0.05. **(B)** Heat map showing the proportion of 22 types of immune cells in high- and low-risk groups. **(C)** Vioplot showing the compression of each immune cell type between high- and low-risk groups. **(D)** Bar plot of PD-L1 expression in high- and low-risk groups.

The tumor-infiltrating immune cells were identified with CIBERSORT. 12 patients were enrolled in this study with the P value of CIBERSORT < 0.05. The infiltrated immunes cells in cholangiocarcinoma are shown in **Figure 9A**. Among 22 kinds of immune cells, M2 macrophage are the main cell types that infiltrate in the cholangiocarcinoma tissue. Compared with low prognostic signature patients, high prognostic signature patients exhibited higher proportion of CD8+ T cells (**Figures 9B, C**). Additionally, the expression of PD-L1 is upregulated in patients with high AS risk score (**Figure 9D**).

## Discussion

The increasing incidence and poor outcomes for cholangiocarcinoma mean that biomarkers for diagnosis and therapy are urgently required. To the best of our knowledge, the biomarkers widely used in the clinic, including carcinoembryonic antigens (CEAs), CA-199, CA-242, and CA-50, have limited cholangiocarcinoma diagnostic and prognostic sensitivity and specify. The technical developments in sequencing techniques, have led to the wide clinical application of genetic diagnosis. Recently, transcriptome signatures have been applied to predict the outcome of cholangiocarcinoma. Wada Y and colleagues constructed a model based on 8 gene expression (BIRC5, CDC20, CDH2, CENPW, JPH1, MAD2L1, NEIL3, and POC1A), which predicts the recurrence of cholangiocarcinoma with AUC of ROC=0.92 (17). Xiaozai Xie and colleagues constructed a model predicting the overall survival of cholangiocarcinoma (AUC of ROC= 0.938) based on 5 lncRNA expression (18). In this study, we additionally provide an effective model based on genomic instability-related AS events for predicting OS with AUC of ROC curve of 0.981.

Genomic instability has been recognized a hallmark of carcinoma genesis. Recent studies have paid attentions to the role of genomic instability in the progression and recurrence indicating that the degree of genomic instability has prognostic implication. Although the molecular mechanisms of genomic instability remain unclear, previous studies have revealed that alternative splicing (AS) are associated with genomic instability (19). Some formula based on alternative splicing signature have been applied to quantify genomic instability degree (20). Recent studies have focused on the AS network, leading to the construction of prognostic signature models based on comprehensive AS events which suitable levels of predictivity and efficiency for carcinoma prognosis (21–23). Whether genomic instability-related AS events could effectively predict prognosis of cholangiocarcinoma remains unclear.

In the present study, we obtained single nucleotide polymorphism data of patients with cholangiocarcinoma from TCGA data sets. We identified differential AS events by comparing patients with genomic stability and those with genomic instability. Then, Univariate Cox regression analysis revealed 26 differential AS events that were associated with the OS in cholangiocarcinoma. K-M survival and ROC analyses showed that this model has robust sensitivity and specify for predicting OS in patients with cholangiocarcinoma. However, a study using a larger cohort is needed to verify the efficiency of our model.

In our prognostic model, we identified the key roles of SLC46A1 and IARS AS in predicting the OS in patients with cholangiocarcinoma. SLC46A1 belongs to solute carrier family and participates in the import of heme folate. Previous studies show that SLC46A1 is abundant in the liver and is responsible for iron metabolism (24). Consistent with our results, Hlavac and colleagues found that SLC46A1 variants are associated with ERBB2/HER2 status and disease-free survival in hormonally treated patients with breast carcinoma (25). The underlying mechanism by which SLC46A1 variants affect cholangiocarcinoma prognosis requires further research.

Isoleucine-tRNA synthetase (IARS) is responsible for aminoacyl tRNA biosynthesis, which plays an essential role in protein translation. Recently, the IARS deficiency has been associated with human disease (26, 27). Hsu and colleagues found that IARS expression is upregulated in oral cavity squamous cell carcinoma (28). Additionally, our results show that IARS-86836-ES variants are associated with poor OS in patients with cholangiocarcinoma. The mechanism underlying this may be associated with insufficient aminoacylation activity to meet translational demand in tumor cells (29).

We then tried to explore the upstream regulators of prognosis associated AS events. Differential expression and hotspot mutations of splicing factor genes have recently been reported in numerous malignancies, suggesting the importance of splicing factors in cancer development and progression. Pearson correlation analysis revealed that CCDC12, CLASRP, CLK4, RBM5, SEC31B, SRSF5, CIRBP, SNRNP70, ZRSR2, PPWD1, CLK1, CDK11A, NOSIP, U2AF1L4, RBM26, HNRNPC, HSPA1B, and CELF2 negatively regulate prognosis associated AS events. Additionally, patients with cholangiocarcinoma were divided into high- and low-expression groups based on splicing factor expression. K-M survival curve analysis revealed that patients with high CDK11A expression levels had higher disease-free survival rates. Consistently, previously studies have reported the tumor-promoting anti-cancer effects of CDK11A (30, 31). Liu and colleagues also found that CDK11A upregulation suppresses cellular proliferation by inducing cell cycle arrest (32).

Recently, immune therapy has emerged as a promising treatment strategy for solid tumors. We summarized previous reported transcriptome signatures related to the change of immune microenvironment in cholangiocarcinoma (**Supplement Table 1**). Michele Ghidini and colleagues have revealed the characterization of the immune-related transcriptome in cholangiocarcinoma (33). They found that high CTLA4 expression, representing the enrichment of Treg cells, in adjacent tissue is associated with the poor recurrence free survival of cholangiocarcinoma. In addition to their study, we analyzed difference of infiltrated immune cells in patients with different alternative splicing signature. We found that the proportion of CD8+ T cells is upregulated in carcinoma tissue of patients with higher splicing signature scores. Ying Zhu and colleagues found that INF-γ secretion by CD8+ T cells may increase cancer cell PD-L1 expression (34). Upregulated PD-L1 on cancer cells has been recognized as a marker of immune escape and poor outcome in patients with cholangiocarcinoma (35). Consistent with this, we found higher levels of PD-L1 expression in the high-risk group. Therefore, the high-risk group may more effectively respond to anti-PD-L1/PD-1 therapy. Finally, we also produced a nomograph model for predicting 1-, 3-, and 5-year survival in patients with cholangiocarcinoma based on age, sex, TMN stage, mutation counts, and risk score.

In conclusion, we have developed a prognostic signature for OS in patients with cholangiocarcinoma based on cancer pathway-related AS events. Additionally, AS events SLC46A1-39899-ES, IARS-86836-ES, and the CDK11A splicing factor may be therapeutic targets for cholangiocarcinoma. Anti-PD-L1/PD-1 immunotherapy may be a promising therapeutic strategy for patients with cholangiocarcinoma and high-risk scores. However, the small sample size used in this study means that our results require further external examination.

## Data Availability Statement

The datasets presented in this study can be found in online repositories. The names of the repository/repositories and accession number(s) can be found in the article/supplementary material.

## Author Contributions

ZZ designed the study and wrote the manuscript. ZL collected data and completed the data analysis. GZ and JH wrote the R script. JG and LZ guided the study and checked the data. All authors contributed to the article and approved the submitted version.

## Funding

This study was funded by the National Natural Science Foundation of China (Grant NO.81970510), Talent Project of Chongqing (CQYC2019050790), and the Graduate Student Innovation Project of Chongqing (Grant number: CYB20153).

## Conflict of Interest

The authors declare that the research was conducted in the absence of any commercial or financial relationships that could be construed as a potential conflict of interest.
