# Supplementary material for: Identification of Mutator-Derived Alternative Splicing Signatures of Genomic Instability for Improving the Clinical Outcome of Cholangiocarcinoma
Source: Front Oncol. 2021 May 14;11:666847. doi: 10.3389/fonc.2021.666847 (PMC8160381; doi:10.3389/fonc.2021.666847)
Supplement: Supplementary file 1 [file DataSheet_1.docx]

| Author,year | PMID | transcriptome signature | effects of transcriptome signature | |
| --- | --- | --- | --- | --- |
| Chen Z,2021 | 33841428 | PNOC and LAIR2 | | PNOC, expressed by B cells, could predict better survival of patients;  LAIR2 expressed by exhaustive T cell populations, correlated with worse survival of patients |
| Huang X,2021 | 33685460 | CD247, FCGR1A, and TRRAP | | correlated with superior prognoses and infiltration of antigen-presenting cells |
| Zhao Y,2020 | 33552963 | CMTM6 | | related to PD-L1 protein expression and increased infiltration of neutrophils |

Supplement table 1
